# Draft Genome Sequence of *Bacillus pumilus* ku-bf1 Isolated from the Gut Contents of Wood Boring *Mesomorphus* sp.

**DOI:** 10.3389/fmicb.2016.01037

**Published:** 2016-06-30

**Authors:** Jatoth Balsingh, Surabhi Radhakrishna, Kandasamy Ulaganathan

**Affiliations:** Center for Plant Molecular Biology, Osmania UniversityHyderabad, India

**Keywords:** *Bacillus pumilus*, genome sequencing, cellulolytic bacteria, xylose isomerase, bioethanol, *Mesomorphus*

## Introduction

The threat of climate change has intensified efforts toward the development of safer alternatives to depleting fossil fuels (Cox et al., 2000). Lignocellulosic bioethanol is considered to be a viable and environmentally friendly alternative to fossil fuels. Though lignocellulosic biomass is available in massive quantities and is renewable (Dillon and Dillon, 2003; Lynd et al., 2008; Pauly and Keegstra, 2008; Kricka et al., 2015), the presence of certain barriers makes lignocellulosic bioethanol expensive. Discovery of proteins with novel specificities is necessary to break these barriers and make lignocellulosic bioethanol economically viable (Horn et al., 2012; Ulaganathan et al., 2015). Cellulolytic bacteria isolated from various environments have been explored for proteins of potential use in lignocellulosic bioethanol production (Badger, 2002; Wang et al., 2012; Pinheiro et al., 2015). Bacteria belonging to the genera *Bacillus, Bacteroides, Butyrivibrio, Cellulosimicrobium, Citrobacter, Clostridium, Devosia, Dyadobacter, Ensifer, Kaistia, Labrys, Methanobrevibacter, Microbacterium, Ochrobactrum, Paracoccus, Pseudomonas, Rhizobium, Ruminococcus, Shinella, Siphonobacter, Stenotrophomonas, Trichonympha*, and *Variovorax*, were found to be cellulolytic (Saxena et al., 1993; Schwarz, 2001; Gupta et al., 2012; Huang et al., 2012; Yanga et al., 2014). *Bacillus pumilus* strains are known to produce cellulase enzyme up to a maximum of 11.4 mg/g of cell dry mass (Suzuki and Kaneko, 1976; Kotchoni and Shonukan, 2002; Ariffin et al., 2006). The cellulase enzyme produced by *B. pumilus* strain EB3 has been found to be superior to fungal cellulases due to its higher optimum pH and temperature (Ariffin et al., 2006). Further it has been shown that the *B. pumilus* cellulase enzyme could be mutated to remove the catabolite repression (Kotchoni et al., 2003). We have recently isolated bacterial strains from the gut contents of the wood boring *Mesomorphus* sp. These isolates were screened for cellulolytic and xylose isomerase activities and the isolate ku-bf1 which exhibited maximum cellulolytic and xylose isomerase activities was identified as *B. pumilus* by 16S rRNA sequencing. The whole genome of this strain has been sequenced. The dataset has been submitted to NCBI and is reported here.

## Materials and methods

### Isolation of the bacterial strain

Bacterial isolates were made by plating the gut contents of wood boring *Mesomorphus* sp. on YEP-Agar medium (Yeast extract, peptone and agar). After incubation for 24 h at 25°C, the growing bacterial colonies were sub-cultured. These colonies were tested for cellulolytic and xylose isomerase activities on CMC-Agar medium (NH_4_H_2_PO_4_—1 g/L; KCl—0.2 g/L; MgSO_4_.7H_2_ O—1 g/L; Yeast Extract—1 g/L; Carboxymethyl Cellulose—26 g/L; Agar—3 g/L) and YEP-Xylose-Agar medium, respectively (Sapunova et al., 2004; Ponnambalam et al., 2011). The bacterial isolate (ku-bf1) which produced maximum clearance zone in both plate assays was selected for this work.

### Genomic DNA isolation, library preparation and sequencing

Genomic DNA was isolated using a modified Cetyltrimethyl ammonium bromide (CTAB) method (Murray and Thompson, 1980; Zhou et al., 1996). The quality of isolated DNA was checked using a Qubit fluorimeter (Thermo Fisher) and 50 ng of pure genomic DNA was used for library preparation. Genomic DNA was fragmented and adapter-tagged using a Sure Select QXTKit (Agilent Technologies). Fragmented DNA was cleaned using HighPrepBeads (MagBio Genomics). Cleaned and adapter tagged fragments were amplified and indexed. The prepared library was quantified using a Qubit Fluorimeter. The quality of the library was checked by running an aliquot (1 ul) on a High Sensitivity Bioanalyzer DNA Chip (Agilent Technologies). The library showed a size range of ~300–1000 bp in the Bioanalyzer profile. The effective insert size of the library was in the range of ~180–880 bp, Whole genome sequencing was carried out with an IluminaMiseq system (Illumina, San Diego, CA) at Genotypic Technology (P) Ltd., Bangalore

### Preprocessing and genome assembly

The quality of sequence reads was analyzed using the FastQC tool (Andrews, 2010). Reads were trimmed off adapters using the Fastx-toolkit (Gordon and Hannon, 2010). Reference genome assembly was carried out using the Bowtie2 tool (ver. 2.2.4) (Langmead and Salzberg, 2012). The genome of *B. pumilus* W3, downloaded from Genbank, was used as the reference genome. Reference based assembly involved indexing of the reference genome and alignment of reads to the reference and creation of a SAM file using SAMtools (ver 0.1.18) (Li et al., 2009). The SAM file was converted to a binary BAM file, sorted and indexed by using the “view,” “sort” and “index” functions of SAMtools, respectively. The BAM file was checked using the BamView tool and used for variation report generation (Carver et al., 2010). The consensus sequence was generated using SAMtools. The variation report in “bcf” format was converted into a “vcf” file using BCFTools.

## Results

### Whole genome sequencing of *B. pumilus* ku-bf1

Sequencing the genome of *B. pumilus* ku-bf1 produced a total of 3,841,334 paired-end reads (150 bp). After removing adapters and low quality reads, the reads were used for reference based genome assembly. These reads were assembled on to the reference genome (*B. pumilus* W3) using Bowtie-2 (Langmead and Salzberg, 2012). Over 90% of the reads were aligned to the reference genome and the coverage was estimated to be >100x. The BAM file was used for generating the variation report using SAMtools with a mapping quality of >30 and read depth of >20 as cutoffs. The consensus sequence generated was 37,45,118 bp long. NCBI Prokaryotic genome annotation pipeline predicted a total of 3430 protein coding genes, 94 RNA coding genes and 56 pseudogenes. The RNA coding genes predicted include seventy tRNA genes, six 5S rRNA genes, seven 16S rRNA genes, six 23S rRNA genes and five non-coding RNA genes (Table 1).

**Table 1:** ***B. pumilus* ku-bf1 genome characteristics and resources**.

**S. No**	**Name**	**Genome characteristics and Resources**
1	NCBI Bioproject ID	PRJNA298672
2	NCBI Biosample ID	SAMN04230746
3	NCBI Genome Accession Number	CP014165
4	Sequence type	Illumina Miseq
5	Total number of Reads	3,841,334
6	Read length	150
7	Overall coverage	>100x
8	Mapped reads	90 %
9	Estimated genome size	3,745,118 bp
10	GC content	41.64%
11	Protein coding genes	3430
12	tRNA coding genes	70
13	rRNA coding genes	19
14	ncRNA coding genes	5
15	Pseudogenes	56

### Direct link to deposited data and information to users

The dataset submitted to NCBI include the assembled consensus sequence of *B. pumilus* ku-bf1 in Fasta format and the Bam file generated by reference based assembly. The genome sequence can be accessed at NCBI using the accession number CP014165. Users can download and use the data freely for research purpose only with acknowledgment to us and quoting this paper as reference to the data.

## Author contributions

Work was planned by KU and executed jointly by KU and JB. SR was associated with isolation of the bacterial strain.

### Conflict of interest statement

The authors declare that the research was conducted in the absence of any commercial or financial relationships that could be construed as a potential conflict of interest.
